# Feynman Kac Reweighted Schrödinger Bridge Matching for Surface-Based Tau PET Harmonization

**Published:** 2026-06-16

**Authors:** Jianwei Zhang, Xinyu Nie, Jiaxin Yue, Yonggang Shi

## Abstract

Tau PET imaging is central to tracking Alzheimer's disease progression, but systematic differences between scanners, protocols, and radiotracers across sites introduce nonbiological variability that inflates biomarker variance, reduces sensitivity to disease effects, and can bias downstream clinical assessments. Harmonization methods aim to remove these site-induced shifts while preserving biologically meaningful signal, yet existing approaches struggle when source and target cohorts differ in subgroup composition, risking conflation of site effects with biological variation such as tau-positivity status. We propose the Feynman Kac Reweighted Schröodinger Bridge Matching (FKRSBM) model to address this problem. Rather than routing data through a Gaussian noise prior as in diffusion-based methods, FKRSBM learns a direct stochastic transport process between source and target distributions via entropy-regularized optimal transport. To enforce biologically consistent transport, FKRSBM incorporates a subgroup-aware endpoint proposal derived from a Feynman Kac reweighting of the reference bridge measure, implemented entirely through stratified importance sampling at the data level and requiring no changes to the underlying bridge-matching solver or network architecture. For surface-based neuroimaging, FKRSBM employs a spherical convolutional backbone operating on cortical meshes to perform vertex-level harmonization. We evaluate the method on tau PET SUVR maps, harmonizing PI-2620 data from the HABS-HD cohort into the AV-1451 domain of ADNI. Compared against ComBat, CycleGAN, a diffusion-based method (DF), and unregularized Diffusion Schröodinger Bridge Matching (DSBM), FKRSBM achieves superior distributional alignment, reduced tau-positivity sign mismatch, stronger APOE subgroup alignment, and improved downstream disease classification performance.

